# *Plocamium cartilagineum* extract induces transcriptional changes associated with cell-cycle disruption in C2 canine mastocytoma cells

**DOI:** 10.3389/fonc.2026.1869639

**Published:** 2026-07-30

**Authors:** Hanane Maghrebi, Greta Mucignat, Fatima Lakhdar, Bouchra Benhniya, Veronica Di Paolo, Samira Etahiri, Francesca Capolongo, Marianna Pauletto, Mery Giantin, Mauro Dacasto

**Affiliations:** 1 Department of Comparative Biomedicine and Food Science, University of Padua, Legnaro, PD, Italy; 2 Laboratory of Marine Biotechnologies and Environment, National Center for Scientific and Technical Research (Morocco) (CNRST) Labelled Research Unit, Department of Biology, Faculty of Sciences, Chouaib Doukkali University, El Jadida, Morocco

**Keywords:** canine mastocytoma, cell cycle regulation, cytotoxicity, *Plocamium cartilagineum*, red algae (Rhodophyta), transcriptomic analysis (RNA-seq)

## Abstract

**Introduction:**

Within an integrative medicine approach that links veterinary and human health, red algae extracts are gaining attention as promising sources of natural bioactive compounds with potential anticancer properties. This study investigates the selectivity of *Plocamium cartilagineum* extract (PCE) against canine cancer cell lines and its transcriptional effects in C2 canine mastocytoma cells.

**Methods:**

Initial cytotoxicity was assessed in C2 canine mastocytoma cells using three assays: Alamar Blue (AB), Neutral Red Uptake (NRU), and Sulforhodamine B (SRB). Selectivity was then investigated using the AB assay in two canine cancer cell lines, C2 and CLBL-1, and two non-cancer canine cell lines, Cf2Th and MDCK. Following the selectivity assessment, the transcriptional responses induced by PCE in C2 cells were investigated by RNA sequencing (RNA-seq) and validated by quantitative PCR (qPCR).

**Results:**

PCE exhibited cytotoxic activity in C2 cells, with IC_50_ values ranging from 42.15 to 77.75 µg/mL across the three assays. In the selectivity assessment, IC_50_ values ranged from 10.69 µg/mL in CLBL-1 cancer cells to 41.46 µg/mL in Cf2Th non-cancer cells. PCE showed preferential cytotoxicity toward cancer cells, with a selectivity index (SI) reaching 3.9 when comparing CLBL-1 cancer cells with Cf2Th non-cancer cells. RNA-seq and qPCR analyses revealed that PCE induced broad transcriptional changes in C2 cells, including the upregulation of *CDKN1A* and the downregulation of genes involved in cell-cycle regulation, such as *CCNB2*, *CDC25*, and *PLK1*. Functional enrichment analyses further identified biological processes associated with cell-cycle regulation, DNA repair-related pathways, cellular stress responses, and metabolism.

**Conclusion:**

PCE exhibited selective cytotoxic activity toward canine cancer cell lines and induced broad transcriptional alterations in C2 mastocytoma cells. These findings support further investigation of red algae as potential anticancer candidates.

## 1 Introduction

Neoplastic diseases are frequent in companion animals, especially dogs. Mast cell tumors (MCTs) are among the most common ones, arising from uncontrolled mast cell proliferation (1, 2). Canine cutaneous MCTs account for up to 11% of skin neoplasms, and remain a major challenge in veterinary oncology due to their prevalence and complex biology (3–6). They are also relevant in comparative oncology because canine MCT and human mastocytosis share key molecular features, supporting dogs as translational models for mechanistic and therapeutic studies (7, 8).

Current treatment options for canine MCT include surgery, chemotherapy, and tyrosine kinase inhibitors (TKIs) such as masitinib and toceranib (4, 9). However, each modality has limitations related to chemoresistance and side effects (10–12). These constraints support continued efforts to identify additional agents with improved therapeutic windows and complementary mechanisms of action (10–13). Over the past few years, innovative drugs have revolutionized cancer treatment strategies by modulating gene expression pathways central to tumor growth and progression (14). Genes controlling cell cycle progression and cell metabolism have been among the most targeted pathways in cancer therapy (14, 15). For example, transcriptome profiling of triple-negative breast cancer cells exposed to doxorubicin revealed upregulation of *TP53* target genes, including *CDKN1A* (p21) and *TP53INP1*, supporting a p53-driven cell cycle arrest program (16). Despite these advances in drugs targeting the cancer transcriptional machinery, these drugs may have long-term side effects (17). This has driven growing interest in alternatives that preserve therapeutic benefits while minimizing adverse effects.

In veterinary oncology, alternative medicine is evolving, with the use of natural products to treat canine cancers. The advantage of using natural bioactive compounds for cancer treatment lies in the potential synergy of multiple compounds within a single extract, where the primary compound may target one or more pathways, while other molecules may influence additional targets or reduce side effects (18, 19). Few studies have highlighted the potential of natural compounds for treating canine tumors and have demonstrated promising anticancer activity across multiple canine tumor types. The extract obtained from *Calotropis procera* demonstrated an antitumor potential against canine osteosarcoma cells and canine mammary tumor. It affected cancer progression by inducing cell cycle arrest and apoptosis. Remarkably, the effect of this extract was selective to cancer cells (20). Recently, Wagle et al. (21) found that different extracts prepared from *Inonotus obliquus* (Chaga mushroom) and marine microalgae synergistically inhibited the growth of various human and canine cancer cells *in vitro*. In two prior studies, Levine et al. (22, 23) showed that green tea, turmeric, and rosemary extracts inhibit proliferation in three primary canine tumor cell lines, including MCT. Turmeric was most active and acted synergistically with rosemary, and this combination was synergistic with cancer drugs such as toceranib and doxorubicin. Interestingly, the extract affected cancer cells selectively and had no significant effect on non-cancerous primary cells.

Marine algae are among the promising alternative treatments for cancer in veterinary oncology (24). They are a rich source of bioactive compounds with antioxidant (25–27) and inflammation-controlling properties (28), indicating their potential for addressing conditions associated with oxidative stress and the regulation of inflammatory reactions (28–30). Such pharmacological properties hold promise for various medical applications and suggest their potential role in cancer therapy (29), offering new avenues for treatment that could complement or enhance the efficacy of existing conventional therapies. The exploration of algal extracts aligns with the broader trend in oncology toward natural, less toxic, and potentially more effective treatment options (31, 32). Recently, the red alga *Sphaerococcus coronopifolius* extract was shown to possess selective anticancer activity, with a greater cytotoxicity on C2 and NI-1 canine MCT cell lines when compared with the normal canine cell lines Cf2Th and MDCK (33).

The algae of the genus *Plocamium*, and particularly *Plocamium cartilagineum*, are widely recognized as rich sources of fatty acids and polyhalogenated monoterpenes (34), a class of secondary metabolites strongly associated with cytotoxic and antiproliferative activities. Multiple studies have demonstrated that halogenated monoterpenes isolated from *P. cartilagineum* exert potent *in vitro* anticancer effects across a broad spectrum of tumor models, including lung, colon, cervical, neuroblastoma, and leukemia cell lines (35–37). Several of these compounds exhibited selective cytotoxicity toward tumor cells, highlighting their potential to target cancer-specific vulnerabilities rather than inducing non-specific toxicity (35, 38). Beyond isolated natural products, crude extracts of *P. cartilagineum* have also shown antiproliferative activity in human hepatocellular (HepG2) and colorectal (Caco-2) carcinoma models (39, 40), reinforcing the biological relevance of the algal metabolome.

Despite the richness of *P. cartilagineum* in anticancer compounds, no study has yet investigated the selective cytotoxic activity of its extracts in canine MCT cells or characterized the associated transcriptional responses. Here, we address this gap by evaluating the selective cytotoxicity of *P. cartilagineum* extract (PCE) and assessing its effects on gene-expression patterns in the C2 canine MCT cell line, thereby extending its relevance within a comparative oncology framework. Through transcriptomic profiling, the present study provides insight into molecular pathways and biological processes associated with the cellular response to PCE exposure.

## 2 Materials and methods

### 2.1 Algal material

The red alga *P. cartilagineum* was collected by hand-picking during March to April 2021 from the Sidi Bouzid coast, El Jadida, Morocco (33°16′09′′ N, 8°30′-8°45′ W). The alga was thoroughly cleaned to remove epiphytes, sediment, organic debris, and macrofauna. Samples were successively rinsed with seawater, tap water, and distilled water. The rinsed thalli were then completely dried at room temperature (25 °C) and crushed to a fine powder, as reported in Mucignat et al. (33). Macroalgal identification was performed using a combination of methods described in previous studies (41). It was performed on freshly collected specimens using taxonomic identification keys. Various resources, including the Determination Key for Common Atlantic Coast Algae, the Species Identification Guide, the International Code of Nomenclature for Algae, Fungi, and Plants (ICN), and AlgaeBase, were used in the identification process.

### 2.2 Preparation of the algal extract

The algal extract was obtained by macerating 50 g of the dried algal powder in a mixture of dichloromethane/methanol (1:1, v/v) over 72 hrs. Then, the algal extract was filtered, and the solvents were removed by rotary evaporation under reduced pressure at 45 °C until a crude extract was obtained, which was stored at 4 °C until needed. The algal extract was dissolved in dimethyl sulfoxide (DMSO; 100%) to obtain the stock solution, then diluted in culture medium to obtain the required concentrations.

### 2.3 Chemical analysis of the algal extract

Transesterification of pre-weighted and air-dried algal extract was executed to analyze its chemical composition through gas chromatography (GC) (GC TRACE 1300 TSQ 8000 evo) coupled to mass spectrometry (MS). Initially, an esterifying mixture used during the transesterification process was prepared. For this mixture, 500 μL of 12% hydrochloric methanol was added to 300 μL of methanol, followed by the addition of 100 μL of chloroform. This preparation was then added to 50 mg of dried algae extracts, and the mixture was refluxed by heating at 80 °C for 5 hrs. After cooling at room temperature, fatty acid methyl esters (FAMEs) were extracted by 500 µL of hexane at room temperature for 10 min, and the process was performed in duplicate. The resulting hexane solution was recovered and centrifuged for 5 minutes at 2000 rpm. After centrifugation, the hexane solution was collected using a microsyringe and then dehydrated. One mg of the obtained extract was solubilized in 1 mL of CH_2_Cl_2_ for GC-MS analysis of FAMEs.

GC-MS analysis of total FAMEs was performed by injecting 1 μL of sample on a TG-5 (30 m × 0.25 mm × 0.25 μm) GC-MS column (Thermo Fisher Scientific, Munich, Germany). The GC run was performed in splitless mode, and for MS detection an electron ionization mode, with an ionization energy of 70 eV, was used. The maximum temperature applied in the run for this column was up to 300 °C. The injector and interface temperature were held at 250 °C and Helium was used as the mobile phase with a constant flow rate of 1 mL/min. The temperature program started at 100 °C and then increased to 180 °C at a rate of 15 °C min^-1^, plateaued for 5 min. The rate was decreased to 5 °C min^-1^ from 180 to 300 °C and kept constant for 10 min. The ionization source temperature was 280 °C, with a mass range at m/z 40–500 in a 1 s cycle in a full scan acquisition mode. The identification of components was based on their mass spectra profiles and their comparison with those of the NIST/EPA/NIH mass spectral library.

### 2.4 Cell culture

Cell culture conditions for C2, Cf2Th, and MDCK cells were the same as previously described by Mucignat et al. (33). The canine large B-cell lymphoma cell line (CLBL-1) included in the present study was obtained from Barbara C. Rütgen (Institute of Immunology, Department of Pathobiology, University of Veterinary Medicine, Vienna, Austria). CLBL-1 cell line was maintained in RPMI-1640 supplemented with 10% FBS, 2 mM A/G, 1% P/S, and 1% NEAA. All cell lines were maintained at 37 °C in a humidified incubator with 5% CO_2_.

### 2.5 *Plocamium cartilagineum* extract (PCE) cytotoxicity against C2 cells

The PCE cytotoxic potential was evaluated in C2 cells using three complementary assays: Alamar Blue (AB), Neutral Red Uptake (NRU), and Sulforhodamine B (SRB), following the workflow reported in Mucignat et al. (33). Cells were exposed for 48 hrs to increasing concentrations of PCE (**Supplementary Table 1**). For each assay, three independent biological replicates were performed, and each concentration was tested in sextuplicate. 0.33% DMSO was used as a vehicle control (CTRL).

### 2.6 Selectivity of PCE against canine tumor cell lines

To evaluate PCE selectivity, the AB assay was used to compare cytotoxicity across canine cancerous (C2 and CLBL-1) and non-cancerous (Cf2Th and MDCK) cell lines under the same exposure conditions (48 hrs) to increasing concentrations of PCE (**Supplementary Table 1**). As described in Mucignat et al. (33), cells were seeded in 96-well plates at optimized densities (C2: 3 × 10–^4^ cells/well; CLBL-1: 10^5^ cells/well; Cf2Th: 1 × 10^4^ cells/well; MDCK: 1.5 × 10^3^ cells/well). Three independent biological replicates were performed for each cell line, with sextuplicate technical replicates per concentration. DMSO (0.33% for C2, Cf2Th, and MDCK cell lines, and 0.17% for CLBL-1) was used as vehicle control (CTRL). The selectivity index (SI) was calculated according to de Oliveira et al. (42).

### 2.7 RNA sequencing workflow

This experimental workflow was performed as previously described (33), but a summary of the experimental procedures relevant to the present work is provided below.

#### 2.7.1 Cell treatment and incubation

To characterize the transcriptional changes induced by PCE, C2 cells were seeded in 6-well plates at a density of 6 × 10^5^ cells per well and exposed for 48 hrs to two sub-cytotoxic concentrations of the PCE extract (16.66 µg/mL, PCE17; 33.32 µg/mL, PCE33), corresponding to 1/3 and 2/3 of the half maximal inhibitory concentration (IC_50_) value obtained by the AB assay. Control cells were treated with the vehicle only (0.33% DMSO; CTRL). Four independent biological replicates were included for each condition.

#### 2.7.2 RNA extraction

At the end of the exposure period, total RNA was isolated as previously reported by Mucignat et al. (43). RNA quality and integrity were assessed before sequencing, and only samples with RNA Integrity Number (RIN) values > 7 were included in downstream analyses.

#### 2.7.3 RNA-seq library preparation and sequencing

RNA-seq library preparation and sequencing were performed by Novogene Biotechnology (Cambridge, UK), following standard Illumina protocols, on an Illumina NovaSeq 6000 platform using a paired-end 150 bp strategy. CTRL libraries (i.e., 4) were previously reported in a stand-alone study investigating the transcriptional effects of Sphaerococcus coronopifolius on C2 cells (33). In the present study, the same libraries were used as the CTRL condition and reanalyzed together with new libraries generated for this study.

#### 2.7.4 RNA-seq data processing and differential gene expression analysis

Bioinformatic processing and DGE analysis were carried out using the same pipeline described in Mucignat et al. (33). In brief, raw reads underwent quality control, adapter trimming, and pseudoalignment to the canine reference transcriptome (ROS_Cfam_1.0, Ensembl release 109). Gene-level counts were obtained and normalized before DGE analysis. Differentially expressed genes (DEGs) were identified using a false discovery rate (FDR) < 0.05 and an absolute log2-fold change (logFC) > 0.58.

#### 2.7.5 Functional enrichment analysis

Functional enrichment analysis of DEGs was performed using Gene Ontology-Biological Process (GO: BP) and Kyoto Encyclopedia of Genes and Genomes (KEGG) databases, as previously described (33).

#### 2.7.6 RNA-seq data validation by qPCR

To validate the RNA-seq expression data, C2 cells were exposed to PCE33 for 48 hrs. Confirmatory quantitative Real Time RT-PCR (qPCR) analysis was performed on a selected set of DEGs representative of the main biological processes identified by the transcriptomic analysis and functional enrichment results. Specifically, genes involved in cell cycle regulation (*PLK1*, *CCNB2*, *CDKN1A*, *TP53INP1*), DNA damage response (*RAD51*), tumor microenvironment modulation (*CXCL13*), stress- and cancer-related signaling pathways (*FOS*, *JUNB*), and cholesterol biosynthesis (*SQLE*) were selected. These genes were chosen because they showed significant differential expression in the RNA-seq dataset and reflected the major biological processes altered following PCE exposure.

The qPCR validation experiments were conducted according to the protocol previously described by Mucignat et al. (33). Briefly, C2 cells were seeded and treated with PCE33, followed by total RNA extraction. cDNA was synthesized from 1 µg of total RNA using the High-Capacity cDNA Reverse Transcription Kit (Thermo Fisher Scientific, Milan, Italy). qPCR amplification was performed using Power SYBR Green PCR Master Mix (Applied Biosystems, Milan, Italy) on a Light Cycler 480 instrument (Roche Applied Science, Monza, Italy), with 2.5 ng of cDNA per reaction. Each target and reference gene were analyzed in two technical replicates across four independent biological replicates.

The relative gene expression levels were calculated using the comparative cycle threshold (ΔΔCt) method (44). Data were expressed as fold changes in PCE-treated cells relative to CTRL samples, which were set to 1. Primer sequences and qPCR assay performance parameters, including amplification efficiency, slope, linear regression coefficient, and dynamic range, are reported in the Supplementary Material of Mucignat et al. (33).

### 2.8 Statistical analysis

Statistical analyses were performed using GraphPad Prism Software (Version 10.5.0, San Diego, CA, USA). Dose-response curves were generated using a non-linear regression [log(inhibitor) versus (vs) normalized response, variable slope]. The IC_50_ and the R^2^ were provided by the software. As to qPCR results, the statistical analysis was performed applying the Mann-Whitney test (PCE33 vs CTRL). For the validation of RNA-seq results, the Spearman correlation analysis was considered.

## 3 Results

### 3.1 Preliminary GC-MS chemical profile of PCE

Fifteen compounds were detected by the GC-MS analysis of PCE (Supplementary Material: **Supplementary Figure 1**, **Supplementary Table 2**). Among them, seven compounds were successfully identified (**Table 1**). The identified compounds eluted between 14.19 and 27.28 min and comprised three fatty-acid methyl esters (methyl tetradecanoate, 9-octadecenoic acid (Z)- methyl ester, and methyl stearate), one chlorinated aromatic nitrogen compound (4-(para-chlorobenzyl)-pyridine) and one brominated aromatic compound (9,10-dibromoanthracene). The remaining eight detected peaks (RT 17.85, 18.20, 18.48, 21.66, 24.47, 24.86, and 25.41 min) could not be assigned under the applied identification criteria and are reported as “unknown” (Supplementary Material: **Supplementary Table 2**).

**Table 1:** Chemically identified constituents of PCE.

Compound	Rt*	Formula	Molecular weight	Fragmentation pattern	NIST reference (Cas number)
Methyl tetradecanoate	14.19	C15H30O2	242	242, 211, 199, 143, 87, 74	124-10-7
4-(para-Chlorobenzyl)-pyridine	16.08	C12H10ClN	203	203, 168, 167, 139, 125	4409-11-4
9-Octadecenoic acid (Z)-, methyl ester	20.65	C19H36O2	296	296, 264, 222, 180, 123, 111, 98, 83, 74, 69, 55, 41, 29	112-62-9
Methyl stearate	20.95	C19H38O2	298	298, 255, 199, 143, 87, 74	112-61-8
9,10-Dibromoanthracene	26.26	C14H8Br2	334	334, 176, 174, 175, 177, 178, 88, 87, 168, 150	523-27-3
Octamethylcyclotetrasiloxane	27.20	C8H24O4Si4	296	281, 265, 207, 191, 133, 73	556-67-2
9-Octadecyne	27.28	C18H34	250	81, 67, 41, 95, 55, 82, 43, 54, 69, 96, 109, 123, 137	35365-59-4

*Rt, Retention time (as min).

### 3.2 PCE cytotoxicity in C2 canine MCT cell line

**Figure 1:**
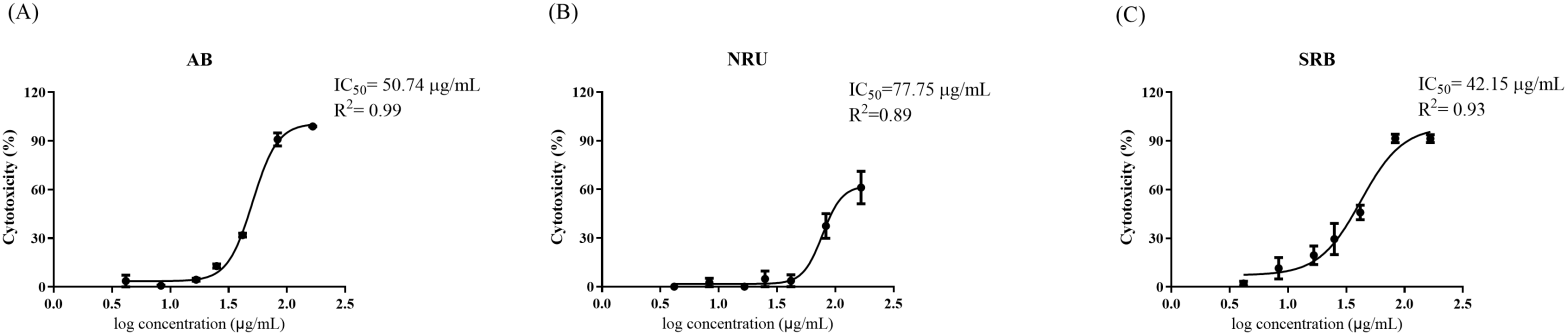
Cytotoxicity (dose-response curves) of *Plocamium cartilagineum* extract (PCE) against C2 canine mast cell tumor (MCT) cell line after 48 hrs exposure, measured by **(A)** Alamar Blue, **(B)** Neutral Red Uptake, and **(C)** Sulforhodamine B assays. Data represent means ± standard error of three independent biological experiments.

The antiproliferative activity of PCE was evaluated in the C2 canine MCT cell line following 48 hrs exposure using three cytotoxicity assays, namely AB, NRU, and SRB. Across all assays, PCE exhibited a concentration-dependent effect, with a typical sigmoidal dose-response profile (**Figures 1A–C**). The resulting IC_50_ values were of the same order of magnitude: 50.74 μg/mL for AB (R² = 0.99), 77.75 μg/mL for NRU (R² = 0.89), and 42.15 μg/mL for SRB (R² = 0.93). Overall, these results are suggestive of a consistent and robust antiproliferative effect of PCE in the canine C2 MCT cell line.

### 3.3 PCE exhibits preferential cytotoxicity toward canine cancer cell lines

The selectivity toward tumor cell lines was assessed using a freshly prepared batch of PCE obtained from the same algal powder and using the same extraction procedure described above. Using the AB assay, cytotoxicity was evaluated in two canine cancer cell lines (C2 and CLBL-1) and two non-cancer canine cell lines (Cf2Th and MDCK). PCE was more toxic toward cancerous cells, as shown by the lower IC_50_ values of 26.57 µg/mL in C2 and 10.69 µg/mL in CLBL-1 compared with 41.46 µg/mL in Cf2Th and 39.20 µg/mL in MDCK (**Table 2**). Selectivity indices (SI), calculated by the ratio IC_50_(non-cancer)/IC_50_(cancer), pointed out a modest selectivity for C2 (SI = 1.6 vs Cf2Th and 1.5 vs MDCK); on the other hand, the selectivity was stronger for CLBL-1 (SI = 3.9 vs Cf2Th and 3.7 vs MDCK) (**Table 3**).

**Table 2:** IC_50_ (µg/mL) of PCE determined by Alamar Blue assay in canine cancer and non-cancer cell lines.

Cells	IC50 (µg/mL)	R2 value
C2	26.57	0.99
CLBL-1	10.69	0.99
Cf2Th	41.46	0.98
MDCK	39.2	0.95

**Table 3:** Selectivity Index (SI) of PCE.

Cancer cell line	SI vs Cf2Th	SI vs MDCK
C2	1.6	1.5
CLBL-1	3.9	3.7

Interestingly, the IC_50_ obtained for C2 in the selectivity experiment (26.57 µg/mL) was lower than that obtained in the initial evaluation of antiproliferative effects (50.74 µg/mL, AB). The only procedural difference between the two extract batches was the solvent evaporation step (nitrogen blowdown using TurboVap LV vs. rotary evaporation), suggesting that evaporation conditions may influence extract composition, leading to measurable differences in cytotoxic potency. Despite this batch-to-batch variation, the PCE used for the selectivity assessment showed a mild to strong SI, particularly against CLBL-1 cells (SI ≈ 3.7-3.9), corresponding to approximately a fourfold higher potency in CLBL-1 cells with respect to non-cancer control cells.

### 3.4 PCE induces transcriptional changes consistent with cell-cycle disruption in MCT cells

**Figure 2:**
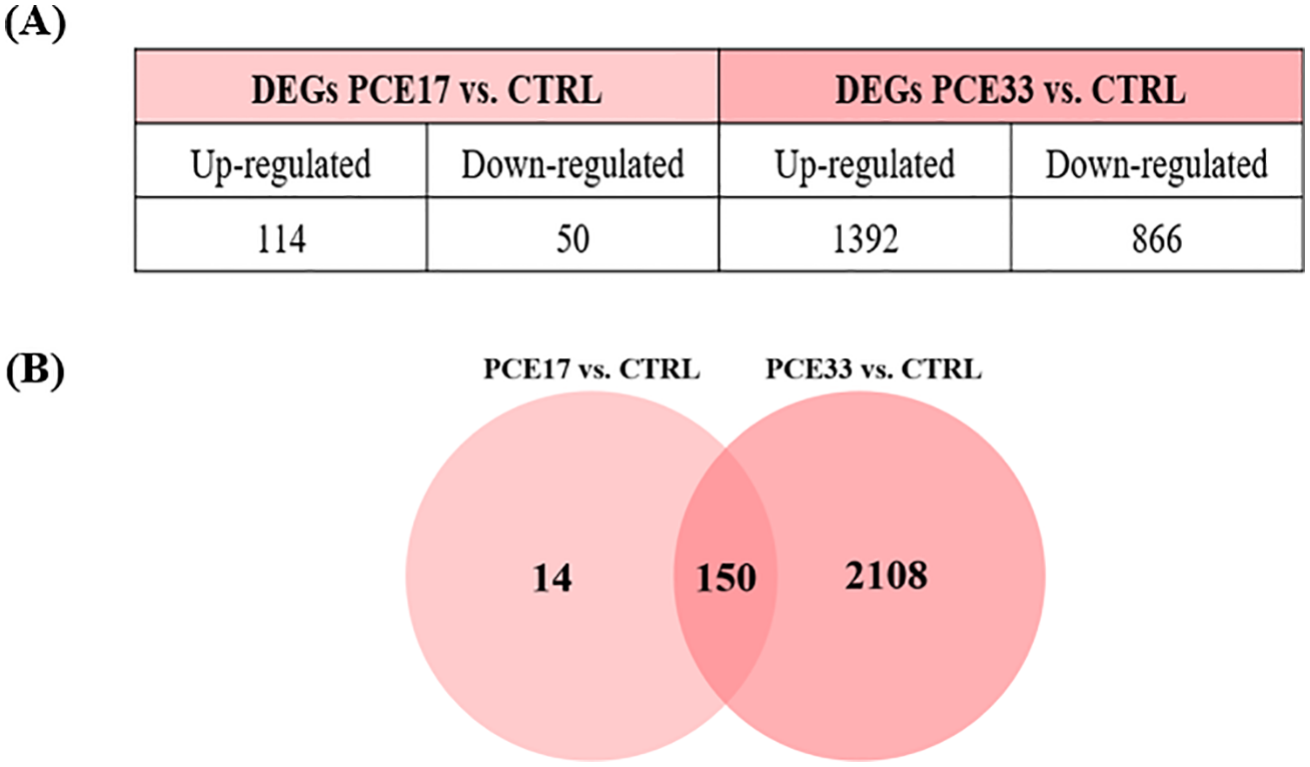
RNA-seq analysis. **(A)** Number of up- and downregulated DEGs obtained comparing PCE17 vs. CTRL and PCE33 vs. CTRL. **(B)** Venn diagram of the shared DEGs in PCE17 vs. CTRL and PCE33 vs. CTRL comparisons. CTRL, control; DEGs, differentially expressed genes; PCE17, *Plocamium cartilagineum* extract, 16.66 μg/mL; PCE33, *Plocamium cartilagineum* extract, 33.32 μg/mL.

For RNA-seq, C2 cells were exposed for 48 hrs to PCE17 and PCE33 (approximately 1/3 and 2/3 of the AB IC_50_, respectively), while cells treated with 0.33% DMSO served as control (CTRL). Sub-cytotoxic concentrations were selected to capture early transcriptional responses preceding extensive cell death. RIN, sequencing, and alignment results are reported in **Supplementary Table 3**, and the complete DGE output is provided in **Supplementary Table 5**. The number of up- and down-regulated DEGs for each condition, together with the Venn diagram of shared DEGs, is shown in **Figure 2**. The number of DEGs increased with PCE concentration, with 164 DEGs at PCE17 and 2,258 at PCE33 (**Figure 2A**). The Venn analysis identified 150 shared DEGs, with 14 DEGs unique to PCE17 and 2,108 unique to PCE33 (**Figure 2B**). Therefore, the main transcriptional response observed at the lower PCE concentration was largely maintained at the higher one, thereby consistent with a concentration-dependent effect. The list of these common DEGs is reported in **Supplementary Table 5**.

Since most DEGs identified at PCE17 were also detected at PCE33, and because the higher dose induced a stronger transcriptional response, we focused the functional enrichment analysis on the up- and downregulated DEGs from the PCE33 vs. CTRL comparison.

In this context, the GO over-representation analysis (**Supplementary Table 6**), performed on the DEGs identified by comparing PCE33 vs. CTRL, revealed 12 and 41 significantly enriched GO terms among up- and downregulated genes, respectively. On the other hand, the KEGG pathway enrichment analysis (**Supplementary Table 6**) identified 21 and 19 significantly upregulated and downregulated pathways, respectively.

**Figure 3:**
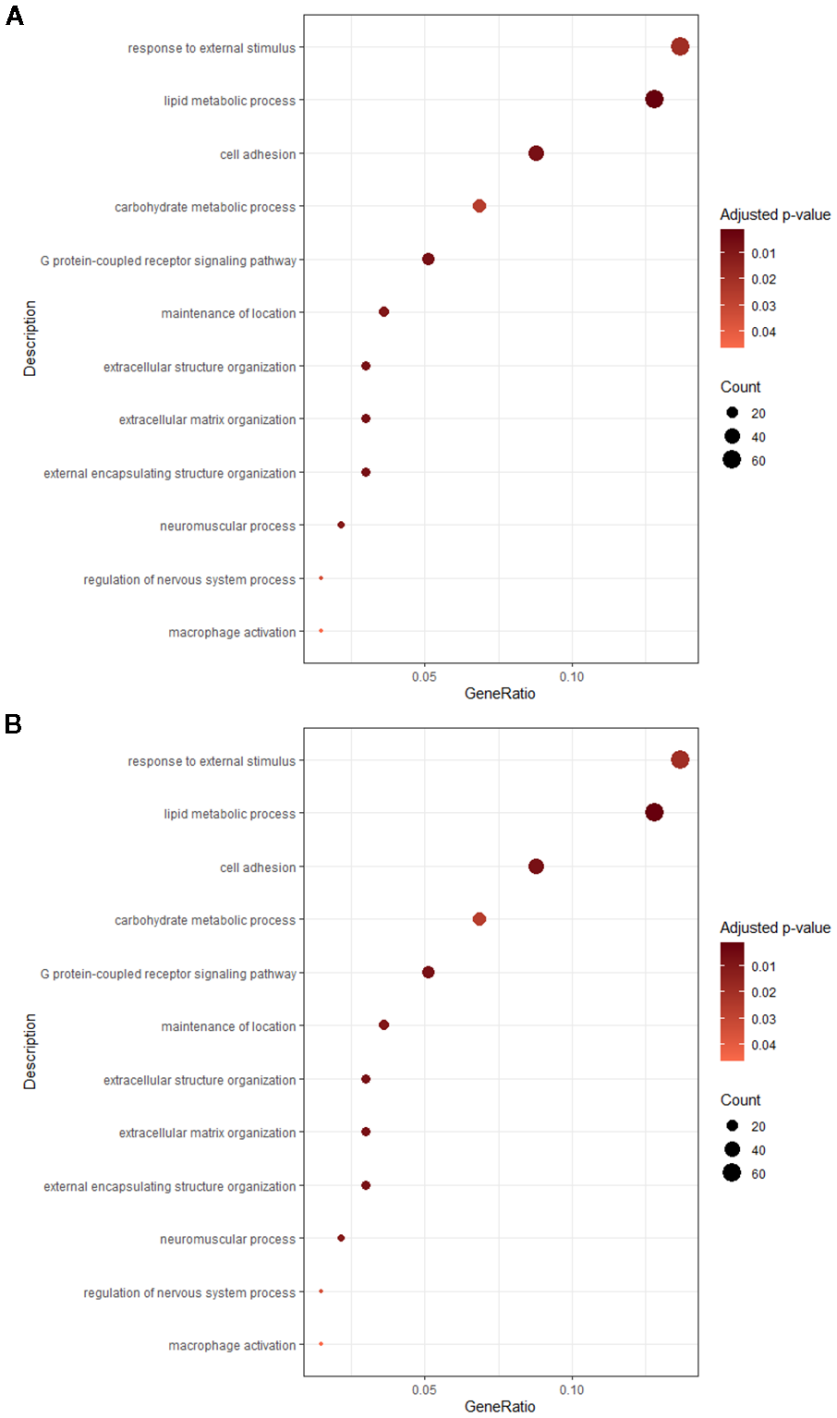
GO enrichment analysis of DEGs (PCE33 vs CTRL). Dot plots show the significant GO terms enriched by **(A)** downregulated and **(B)** upregulated DEGs. The dot size indicates the number of genes contributing to each term. The color gradient is related to the level of significance, adjusted with the Benjamini-Hochberg (BH) method. BH, Benjamini-Hochberg; CTRL, DMSO control; GO, Gene Ontology; DEGs, differentially expressed genes; PCE33, *Plocamium cartilagineum* extract (33.33 μg/mL).

The GO biological processes enriched by downregulated genes (**Figure 3A**) revealed a strong predominance of pathways related to cell cycle, cell division, and spindle checkpoint signaling (GO:0007049; GO:0051301; GO:0051293; GO:0051653; GO:0031577; GO:0071173). Processes associated with chromosome organization and segregation, as well as nuclear division, were also significantly enriched (GO:0051276; GO:0007059; GO:0051985; GO:0000280). DNA replication and DNA metabolic processes (GO:0006260; GO:0006259) are worth mentioning, too.

The key genes contributing to the enrichment of these pathways include Polo-like kinase 1 (*PLK1*); cyclins involved in cell cycle progression like Cyclins A2 (*CCNA2*), B1 (*CCNB1*), B2 (*CCNB2*), and B3 (*CCNB3*); mitotic regulators such as Aurora Kinase B (*AURKB*); and genes involved in DNA damage response and chromosome dynamics, including RAD51 recombinase (*RAD51*), H2A.X variant histone (*H2AX*), NDC80 kinetochore complex component (*NDC80*), and Cell Division Cycle-Associated proteins 8 and 20 (*CDCA8* and *CDC20*).

Interestingly, although involving a smaller number of genes, cholesterol-associated metabolic pathways were also significantly modulated. These included sterol metabolic and biosynthetic processes (GO:0016125; GO:0016126), together with processes related to secondary alcohol metabolism and biosynthesis (GO:1902652; GO:1902653). While cholesterol metabolism was less prominent than cell cycle-associated pathways in terms of gene counts, a consistent set of key genes contributing to all these enriched processes is therefore worth mentioning. These genes include Squalene Epoxidase (*SQLE*), 24-Dehydrocholesterol Reductase (*DHCR24*), 3-Hydroxy-3-Methylglutaryl-CoA Synthase 1 (*HMGCS1*), Sterol Regulatory Element-Binding Transcription Factor 1 (*SREBF1*), 7-Dehydrocholesterol Reductase (*DHCR7*), Mevalonate Kinase (*MVK*), and NAD(P)-dependent Steroid Dehydrogenase-Like (*NSDHL*).

The KEGG enrichment analysis also identified enrichment of pathways related to cell cycle and steroid biosynthesis in PCE33-exposed cells (**Supplementary Figure 2**). In particular, significant downregulation was observed for the “Cell cycle” (cfa04110) and “DNA replication” (cfa03030) pathways, as well as for metabolic pathways including “Steroid biosynthesis” (cfa00100) and “Terpenoid backbone biosynthesis” (cfa00900). In addition, pathways involved in RNA processing and genome maintenance, such as “Spliceosome” (cfa03040) and “Base excision repair” (cfa03410), were also negatively enriched.

On the other hand, the GO biological processes enriched by upregulated genes (**Figure 3B**) revealed a strong predominance of pathways related to lipid and carbohydrate metabolism (GO:0006629; GO:0005975) and cell adhesion (GO:0007155). KEGG over-representation analysis (**Supplementary Figure 2**) supported these findings and identified similar enrichment (cfa04514, cfa04518, cfa00565) and further suggested the modulation of cAMP signaling (cfa04024) and inflammation-related pathways (cfa04060), along with cancer-related pathways (cfa05200, cfa04014, cfa04630).

Among the genes that are contributing to lipid metabolism, there are Peroxisome Proliferator Activated Receptor Alpha (*PPARA*), Carnitine Palmitoyl Transferase 1A (*CPT1A*), the fatty acid homeostasis modulator ARV1 homolog, Delta 4-Desaturase Sphingolipid 2 (*DEGS2*), Glycerol-3-Phosphate Acyltransferase 3 (*GPAT3*), and Solute Carrier Family 27 Member 1 (*SLC27A1*). Regarding the carbohydrate metabolism pathway, the Ribokinase (*RBKS*) and Solute Carrier Family 5 Member 3 (*SLC5A3*) are among the main candidate genes. Other important genes were dysregulated in both lipid and carbohydrate pathways; they include Pyruvate Dehydrogenase Kinase 2 and 4 (*PDK2* and *PDK4*) as well as Hexose-6-Phosphate Dehydrogenase/Glucose 1-Dehydrogenase (*H6PD*).

Among the upregulated genes contributing to the cell adhesion pathway, key structural and signaling components were identified; these included Cadherins 1, 3, and 13 (*CDH1*, *CDH3*, and *CDH13*), together with Catenin Alpha 3 (*CTNNA3*) and Tight Junction Protein 3 (*TJP3*).

Among DEGs positively modulated in PCE33-exposed cells, several genes associated with cancer development were identified, including the proto-oncogenes *FOS*, *JUN*, and *JUNB*, as well as Cyclin-Dependent Kinase Inhibitor 1A (*CDKN1A*) and Tumor Protein p53-Inducible Nuclear Protein 1 (*TP53INP1*).

### 3.5 RNA-seq data validation by qPCR

**Figure 4:**
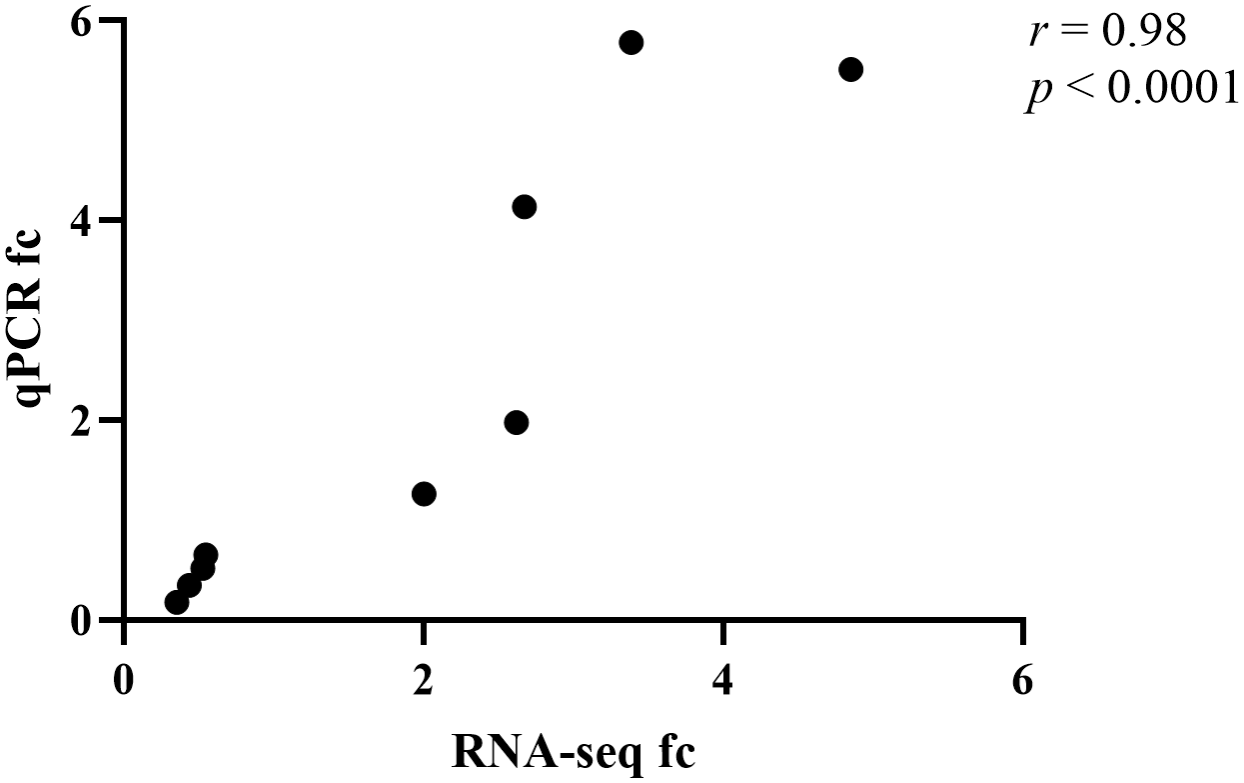
Correlation analysis of RNA-seq and qPCR mRNA levels. The dot plot shows the Spearman's correlation coefficient (r) and the resulting level of significance obtained when comparing the RNA-seq and qPCR mRNA levels (in terms of fc) of 9 target DEGs identified following the RNA-seq analysis. DEGs, differentially expressed genes; fc, fold changes; qPCR, quantitative RT-PCR.

To validate the RNA-seq data, total RNA extracted from C2 cells incubated with PCE33 was used to run confirmatory qPCR analysis on a selected number of DEGs. These targets included cell cycle and DNA repair-related genes (*RAD51*, *CCNB2*, and *PLK1*), lipid metabolism-associated genes (*SQLE*), and cancer progression-related genes (*CDKN1A*, *CXCL13*, *FOS*, *JUNB*, and *TP53INP1*). The obtained qPCR results largely confirmed the RNA-seq findings (**Figure 4**; **Supplementary Table 4**). Genes identified as downregulated by RNA-seq, including *RAD51*, *CCNB2*, *PLK1*, and *SQLE*, exhibited a similar behavior in qPCR analysis, confirming the consistency of the observed transcriptional changes following PCE33 exposure. At the same time, genes upregulated in the transcriptomic analysis, such as *CDKN1A*, *FOS*, *TP53INP1*, and *JUNB*, were found to be similarly upregulated by qPCR. These results confirm the RNA-seq-derived transcriptional modulation of genes associated with cell-cycle regulation, cellular stress responses, DNA repair-related pathways, and lipid metabolism. Taken together, the strong consistency (*Spearman r* = 0.98, *p* < 0.0001) between RNA-seq and qPCR results confirms the reliability of the RNA-seq dataset and the reproducibility of the observed gene-expression changes.

## 4 Discussion

*P. cartilagineum* is widely recognized as a chemically rich red alga that produces a variety of bioactive compounds, including fatty acids and polyhalogenated monoterpenes. These compounds are commonly associated with *in vitro* anticancer activity (36, 37). Here, we present the first investigation of the cytotoxic effects of PCE on the C2 canine MCT cell line, extending prior work that has largely focused on human tumor models. Furthermore, we elucidated the transcriptional modulation behind its potential anticancer activity.

The PCE chemical profile shows that the algal extract contains both C18 and C14 fatty acids (appearing as C19 and C15 species after methylation). This suggests that *P. cartilagineum* lipids in this sample contain a range of long- and medium-chain fatty acids. Concerning the saturation state, two saturated compounds and one monounsaturated compound were identified. This diversity of methylated fatty acids supports the idea that both membrane-associated lipids and storage lipids contribute to the overall chemical profile of the extract (45, 46). The fatty acid profile is relevant to both the bioavailability and the bioactivity of the extract, because lipid constituents can modulate membrane biophysical properties and cellular lipid signaling (46–48). Indeed, the balance between saturated and unsaturated fatty acids can influence uptake and intracellular distribution, membrane integrity, and stress-responsive pathways (46, 48). These effects may influence the cytotoxic responses in non-malignant and malignant cells, including the selectivity of the extract toward cancer cells (49, 50). Because the extract was subjected to transesterification before GC-MS analysis, the resulting chromatographic profile was expected to be enriched in fatty acid methyl esters and may underrepresent or chemically alter other classes of secondary metabolites, including halogenated monoterpenes. A recent study by Ko et al. (34), which also performed GC-MS profiling, has revealed a composition dominated by fatty acids and lipid-like compounds, with palmitic acid as a major constituent, along with a limited number of additional bioactive molecules.

It is meaningful to highlight the challenges related to the *P. cartilagineum* protocol of extraction and the resulting biological effects (*e.g.*, cell sensitivity) observed in the present study. Indeed, variations in IC_50_ values measured in C2 cells were noticed following modifications of the extraction protocol. The only procedural difference was the solvent-evaporation step, which is known to influence extract composition and consequently, its biological activity. Consistent with this observation, Bennour et al. (51) demonstrated that changing the evaporation method for the *Moringa oleifera* hydro-methanolic extract results in shifts in both phenolic profiles and antioxidant activity, likely driven by differences in oxygen and light exposure, evaporation time, and temperature- or pressure-related effects on labile compounds. We therefore acknowledge that the lack of complete standardization of the entire extraction workflow represents a limitation of the present study and may have contributed to the variability observed in cytotoxic activity. As highlighted by Sun et al. (52), even minor changes in extraction or post-extraction conditions can affect phytochemical composition and bioactivity, underscoring the importance of rigorous extraction standardization. Accordingly, future studies should employ fully standardized extraction procedures to ensure reproducibility and comparability between extract batches.

Despite the effect of the extraction procedure on the IC_50_ values obtained in C2 cells, PCE exhibited a mild to strong selectivity toward cancer cells, with higher SI values for CLBL-1 cells (SI ≈ 3.7-3.9). The observed selective cytotoxicity of PCE against canine tumor cell lines is consistent with previous reports on *P. cartilagineum*-derived metabolites. In particular, de Inés et al. (35) demonstrated how several halogenated monoterpenes isolated from *P. cartilagineum* showed preferential cytotoxic effects on tumor cell lines compared with non-tumor mammalian cells, thus supporting the hypothesis that *Plocamium*-derived compounds can selectively target cancer cells.

Starting from these evidences, the present study takes a step further by investigating the transcriptional programs modulated by PCE. In this context, an RNA-seq analysis was performed on C2 cells exposed to two sub-cytotoxic PCE concentrations (PCE17 and PCE33, corresponding to 1/3 and 2/3 of the IC_50_, respectively). The analysis revealed a clear concentration-dependent transcriptional response. The pronounced increase in the number of DEGs at PCE33, together with the substantial overlap with those identified at PCE17, supports a progressive transcriptional remodeling driven by increasing extract concentration. Exposure to PCE33 induced a broad and robust transcriptional response in C2 cells characterized by profound modulation of genes and pathways primarily associated with cell cycle regulation, DNA replication and repair, metabolic dysregulation, and cellular stress responses. In particular, the most significantly enriched terms, obtained from the downregulated DEGs, were dominated by cell cycle and mitotic processes, including chromosome organization and segregation, nuclear division, and DNA replication. This pattern suggests coordinated repression of transcriptional programs required for proliferation and genome duplication. Such transcriptional downshifting of proliferation-associated programs aligns with the cytotoxic and antiproliferative phenotype observed *in vitro*, suggesting that PCE33 inhibited C2 cell growth by mainly interfering with the gene network required for cell cycle progression.

The transcriptomic data of the present study collectively support the hypothesis that exposure of MCT cells to PCE induces a coordinated cascade of transcriptional changes converging on cell cycle and metabolism perturbations. Indeed, the observed induction of *CDKN1A* (coding for p21) together with the repression of core regulators that license the G2/M transition and mitotic progression, including *CCNB2*, *CDC25*, and *PLK1*, could be linked to cell cycle disruption. The upregulation of *CDKN1A* here observed aligns with previous studies showing that certain compounds derived from marine algae can induce p21 expression and, consequently, inhibit cancer cell proliferation. For instance, PPM1, a synthetic analog of polyhalogenated monoterpenes from the red alga *Plocamium*, has been shown to trigger p21 accumulation in breast cancer cells, thereby reducing cell viability (53). p21 generally restrains CDK-dependent phosphorylation pathways that drive cell cycle progression, thereby favoring cell cycle arrest (54). Indeed, *CCNB2, CDC25* and *INCA1*, expected to reduce the activity of cyclin-CDK1 and cyclin-CDK2 complexes (55–60), appeared modulated in this study. An additional and highly consequential layer to this cascade of events, reinforcing the hypothesis of PCE-mediated perturbation of cell cycle-related genes, was finally given by the downregulation of *PLK1*. Interestingly, natural compounds such as DHMMF, isolated from *Resina Draconis*, have been shown to induce DNA damage, G2/M arrest, and apoptosis through p21 upregulation, with *PLK1* acting as a critical downstream effector of this response (61).

The present study also showed that the algal extract caused a downregulation of key drivers of genome maintenance in C2 MCT cells, including *RAD51* and *H2AX*. *RAD51* is a mediator of homologous recombination repair; its high expression has been associated with more aggressive canine MCT phenotypes (62, 63). Decreased *RAD51* mRNA expression is expected to impair the efficient repair of DNA double-strand breaks, maintaining DNA damage signaling and p21 activity in tumor cells exposed to the algal extract. PCE also induced *TP53INP1*, a p53-inducible stress-response gene. The *TP53INP1* upregulation is potentially associated with the activation of a DNA damage-driven stress response centered on the p53 signaling network (64). Importantly, *TP53INP1* is frequently downregulated or silenced in aggressive cancers, and its re-expression has been shown to suppress tumor growth, promote apoptosis, induce autophagic cell death, and limit migratory capacity (65).

Beyond the transcriptional changes associated with cell-cycle regulation and DNA repair-related pathways, PCE exposure also affected the expression of genes involved in lipid and carbohydrate metabolism. The modulation of genes such as *PPARA*, *CPT1A*, *GPAT3*, *DEGS2*, *PDK2*, *PDK4*, *RBKS*, and *H6PD*, together with the enrichment of metabolism-related GO terms and KEGG pathways, suggests that metabolic processes could be involved in the cellular response to PCE exposure. Similar metabolism-associated transcriptional effects have been reported by Begolli et al. (66) in colon cancer cells exposed to red seaweed extracts. Given the importance of metabolic adaptations in supporting cancer-cell growth, survival, and stress tolerance (67), the modulation of metabolism-related genes and pathways observed in our study may provide additional insight into the transcriptional response of C2 cells to PCE exposure.

In addition, the KEGG analysis identified the cAMP signaling pathway among those activated. This enrichment was supported by the modulation of several pathway-associated genes such as the adenylate cyclases *ADCY4* and *ADCY6*, as well as early response genes like *FOS* and *JUNB* and members of the CREB family of transcription factors (*CREBL2*, *CREB3L2*, and *CREB3L3*). These latter transcription factors are of particular interest because they connect cellular stress responses and metabolic regulation (68). Together, these observations suggest that the cAMP-related signaling pathway may contribute to the cellular stress-response induced by PCE exposure. However, as the present study relied exclusively on transcriptomic analyses, the functional significance of these transcriptional changes remains to be elucidated more in depth through dedicated metabolic and biochemical assays.

Overall, PCE demonstrated mild to strong selectivity against canine tumor cell lines and, in the C2 cell line, induced transcriptional alterations in pathways related to cell-cycle regulation, cellular stress responses, DNA repair, and metabolism. However, the present study has some limitations that should be considered. Although RNA-seq and qPCR analyses provided a comprehensive overview of the transcriptional responses induced by PCE in C2 MCT cells, further validation is required. To do so, protein-level confirmatory assays and the inclusion of multiple time points (beyond 48 hrs) could help to confirm the mechanism underlying PCE effects and fully capture the temporal dynamics of molecular responses. Moreover, given that this is an *in vitro* study in which a crude extract was used, the identification and isolation of the bioactive compounds primarily responsible for its biological activity, as well as their preclinical validation in appropriate animal models, should be considered.

Despite these limitations, the present findings support the potential of *P. cartilagineum* for anticancer therapy in canine MCT and warrant further investigation. As natural products continue to attract interest as a source of bioactive molecules for drug discovery, biodiversity conservation, and sustainable resource management also remain important considerations. *P. cartilagineum* is widely distributed in temperate marine areas of the Atlantic Ocean, the Mediterranean Sea, and other coastal regions worldwide (69, 70). Although the specie is not currently listed as threatened by international conservation authorities, natural populations may be affected by local anthropogenic pressures, including coastal pollution, habitat degradation, and climate change. Therefore, any large-scale exploitation for pharmaceutical purposes should be accompanied by sustainable harvesting practices, cultivation strategies, and monitoring programs to minimize potential ecological impacts and ensure the long-term conservation of local populations and ecosystem sustainability.

## 5 Conclusion

Natural compounds, such as those derived from marine algae, are an appealing source of molecules for anticancer therapy. In this context, *P. cartilagineum* has already been reported as a source of bioactive molecules. In the present study, we performed a preliminary *in vitro* assessment of the activity of its dichloromethane/methanol extract on a canine MCT cell line. This work represents an initial step in investigating the mechanisms underlying the potential anticancer activity of PCE, which appears to induce transcriptional perturbations in cell-cycle, cellular stress, DNA repair, and metabolism-associated genes. Even though limited to the transcriptional level, these findings provide the foundation for future studies, in which transcriptomic profiling should be integrated with functional and biochemical assays to validate the biological relevance of the pathways here identified.

## Data Availability

Raw Illumina sequencing data have been deposited in GenBank (SRA) under the BioProject accession PRJNA1287075.
