# Supplementary material for: Plocamium cartilagineum extract induces transcriptional changes associated with cell-cycle disruption in C2 canine mastocytoma cells
Source: Front Oncol. 2026 Jul 30;16:1869639. doi: 10.3389/fonc.2026.1869639 (PMC13468921; doi:10.3389/fonc.2026.1869639)
Supplement: Supplementary file 1 [file Table3.docx]

Supplementary Material

# 1 Supplementary Figures

**Supplementary Figure 1.** Chromatogram obtained from the GC-MS analysis of the derivatized extract of *Plocamium cartilagineum*.

| **(A)** |  | |
| --- | --- | --- |
| **(B)** |  |  |

**Supplementary Figure 2.** KEGG enrichment analysis of DEGs (PCE33 vs CTRL). Dot plots show the significant terms enriched by (A) downregulated and (B) upregulated DEGs. The dot size represents the number of genes belonging to each pathway. The colour gradient is related to the level of significance, adjusted with the Benjamini–Hochberg (BH) method. BH, Benjamini–Hochberg; CTRL, DMSO control; KEGG, Kyoto Encyclopedia of Genes and Genomes; DEGs, differentially expressed genes; PCE33, *Plocamium cartilagineum* extract (33.32 μg/mL).

# 2 Supplementary Tables

**Supplementary Table 1.** Concentrations of PCE (μg/mL) used for cytotoxicity and selectivity assessment.

| Dose level | C2 | CLBL-1 | Cf2Th | MDCK |
| --- | --- | --- | --- | --- |
| 1 | 4.16 | 2.08 | 10 | 5 |
| 2 | 8.33 | 4.16 | 20 | 10 |
| 3 | 16.67 | 8.33 | 30 | 20 |
| 4 | 25 | 16.67 | 40 | 30 |
| 5 | 41.66 | 25 | 50 | 40 |
| 6 | 83.33 | 41.66 | 100 | 50 |
| 7 | 166.67 | 83.33 | 200 | 100 |

**Supplementary Table 2.** GC-MS derivatized extract of *Plocamium cartilagineum*.

| Peak | **Rt^a^** | **Name^b^** | **Formula** | **Molecular weight** | **Fragmentation pattern** | **NIST reference**  **(Cas number)** |
| --- | --- | --- | --- | --- | --- | --- |
| 1 | 14.19 | Methyl tetradecanoate | C_15_H_30_O_2_ | 242 | 242, 211, 199, 143, 87, 74 | 124-10-7 |
| 2 | 16.08 | 4-(para-Chlorobenzyl)-pyridine | C_12_H_10_ClN | 203 | 203, 168, 167, 139, 125 | 4409-11-4 |
| 3 | 17.85 | Unknown | - | - | - | - |
| 4 | 18.20 | Unknown | - | - | - | **-** |
| 5 | 18.48 | Unknown | - | - | - | - |
| 6 | 20.65 | 9-Octadecenoic acid (Z)-, methyl ester | C_19_H_36_O_2_ | 296 | 296, 264, 222, 180, 123, 111, 98, 83, 74, 69, 55, 41, 29 | 112-62-9 |
| 7 | 20.95 | Methyl stearate | C_19_H_38_O_2_ | 298 | 298, 255, 199, 143, 87, 74 | 112-61-8 |
| 8 | 21.66 | Unknown | - | - | - | - |
| 9 | 22.88 | Simetryn | C_8_H_15_N_5_S | 213 | 213, 170 | 1014-70-6 |
| 10 | 24.47 | Unknown | - | - | - | - |
| 11 | 24.86 | Unknown | - | - | - | - |
| 12 | 25.41 | Unknown | - | - | - | - |
| 13 | 26.26 | Anthracene, 9,10-dibromo- | C_14_H_8_Br_2_ | 334 | 334, 176, 174, 175, 177, 178, 88, 87, 168, 150 | 523-27-3 |
| 14 | 27.20 | Cyclotetrasiloxane, octamethyl- | C_8_H_24_O4Si_4_ | 296 | 281, 265, 207, 191, 133, 73 | 556-67-2 |
| 15 | 27.28 | 9-Octadecyne | C18H34 | 250 | 81, 67, 41, 95, 55, 82, 43, 54, 69, 96, 109, 123, 137 | 35365-59-4 |

* Rt: Retention time (as min).

**Supplementary Table 3.** RIN, sequencing and mapping results of C2 cells exposed to PCE17 and PCE33 for 48 hrs

| Sample name | RIN | Nr. raw reads | Nr. reads survived after trimming | Nr. reads pseudoaligned |
| --- | --- | --- | --- | --- |
| C2_CTRL_1 | 9.9 | 20,979,499 | 20,591,693 | 16,928,571 |
| C2_CTRL_2 | 9.8 | 24,865,458 | 24,362,084 | 19,931,311 |
| C2_CTRL_3 | 9.9 | 22,888,724 | 22,374,059 | 18,200,702 |
| C2_CTRL_4 | 9.9 | 21,967,099 | 21,616,735 | 17,835,794 |
| C2_PCE17_1 | 9.9 | 19,654,213 | 19,326,841 | 15,755,929 |
| C2_PCE17_2 | 9.9 | 21,358,721 | 20,956,081 | 17,113,326 |
| C2_PCE17_3 | 9.8 | 20,776,568 | 20,411,768 | 16,744,659 |
| C2_PCE17_4 | 9.9 | 23,914,338 | 23,541,175 | 19,262,452 |
| C2_PCE33_1 | 9.8 | 20,292,956 | 19,966,171 | 16,057,187 |
| C2_PCE33_2 | 9.8 | 22,710,108 | 22,381,059 | 18,083,152 |
| C2_PCE33_3 | 9.8 | 19,717,206 | 19,391,532 | 15,767,469 |
| C2_PCE33_4 | 9.8 | 23,493,073 | 22,992,263 | 18,709,520 |
| Mean | 9.85 | 21,884,830 | 21,492,622 | 17,532,506 |

CTRL, control; PCE17, *Plocamium cartilagineum* extract, 16.33 μg/mL; PCE33, *Plocamium cartilagineum* extract, 33.32 μg/mL; RIN, RNA integrity number

**Supplementary Table 4.** Mean fold change (fc) of selected DEGs obtained by RNA-seq and qPCR in C2 cells treated with PCE33

| Genes | RNA-seq fc | qPCR fc |
| --- | --- | --- |
| *RAD51* | 0.546 | 0.653 |
| *CCNB2* | 0.526 | 0.517 |
| *CDKN1A* | 2.671 | 4.138 |
| *CXCL13* | 2.001 | 1.265 |
| *FOS* | 4.851 | 5.512 |
| *PLK1* | 0.434 | 0.348 |
| *SQLE* | 0.353 | 0.175 |
| *TP53INP1* | 3.384 | 5.782 |
| *JUNB* | 2.619 | 1.977 |
